# Intubation1Read before the Roswell Park Medical Club.

**Published:** 1893-10

**Authors:** George F. Cott

**Affiliations:** Instructor in Laryngology, University Dispensary, Buffalo, N.Y.; 560 Michigan Street


### Original Communications.

### INTUBATION.[1]

By GEORGE F. COTT, M. D.,
Instructor in Laryngology, University Dispensary, Buffalo, N.Y.

The object of this paper is not to produce something new, but principally to call your attention to some important features of intubation, together with the experience of different operators.

I must necessarily confine my remarks to acute affections of the larynx in children requiring intubation, and will incidentally allude to its practicability in laryngeal stenosis of the adult.

I will not take up your valuable time with an historical sketch of intubation, since every practitioner is more or less familiar with it; it is sufficient to state that the O'Dwyer tubes are the only practicable ones for general use at present, giving by far the most satisfactory results, and bidding fair to hold first place indefinitely. In the following remarks, therefore, I will consider their use only.

The question, whether a child would not do as well if left alone, is pertinent and deserves some notice. I do not always consider the operation one of necessity as regards the saving of life, but principally to relieve obstructive breathing. No doubt, many children in whose larynx a tube had been inserted, would recover without it, but the agony caused by the want of air, before the child becomes delirious, must be fearful. I have been called upon to intubate when the previously attending physicians said "The patient may die or may recover. I have seen some do one and some the other;" but did not advise intubation to relieve the most urgent symptom, obstructive breathing. Since jotting down these notes, I have been talking with other medical men, and have learned that many consider relieving stenosis to be entirely a secondary matter and has no bearing whatever upon the final result. In my opinion this is a grave mistake. Any operator can trace recoveries directly to this mechanical intervention. Even though the child dies, the parents are very much relieved when they know the little patient has not suffocated. If you take into consideration the suffering a child endures, no rest, no sleep for

1. Read before the Roswell Park Medical Club.

two or three days, what a relief it was to find in every instance the little patient fast asleep before you have cleaned the instruments and left the house. I, therefore, maintain that in all cases of laryngeal stenosis, pseudo membrane or no pseudo membrane, if life is threatened, insert a tube, and if the child dies, it will certainly die happy. Then again, the tube serves another purpose. In diphtheria, for instance—for in that disease principally we are called upon to intubate—when the membrane does not extend to the bronchi, the pressure exerted upon the surface surrounding the tube causes maceration of the pseudo membrane and its consequent expulsion by coughing, thereby preventing its active absorption. The argument advanced by those not inclined to interfere, that dyspnea is often observed after as well as before operating because the membrane rapidly extends into the trachea and bronchi, holds good in a limited number of cases only. Dr. O'Dwyer has examined a large number of cases post-mortem, in which stenosis was marked, even though the tube was still in the larynx; he found great swelling of mucous and submucous tissue, the false membrane playing an unimportant part in the obstruction.

A peculiar relaxation of tissue occasionally occurs after a tube has been extracted. Breathing may go on easily for some minutes, when suddenly stenosis recurs, requiring the immediate replacement of the tube or death will speedily follow. In fact, the time may be so short that it is always necessary to have a second tube threaded ; if this has been neglected, a thread may be wound around the upper end and immediately under the collar, and the tube rapidly introduced. The cause of this second obstruction occurring when the child has apparently recovered, and after three or four days the tube removed, cannot possibly be due to reformation of pseudo membrane, as that cannot develop so quickly, but seems to be caused by the relaxed tissue, and possibly the vocal cords having been pressed apart for several days, suddenly collapse, and thus shut off the air. I have had one death follow this accident. The child, four years old, had the tube removed the third and fifth days, each time replacement became necessary within an hour ; the seventh day, while playing about the room, and eating bread, some crumbs probably having been drawn into the tube, it was coughed up, and as immediate assistance could not be had, the child died from suffocation.

We have a habit of counseling patients that in case the tube is coughed up, not to swallow it. This seems to be a needless precaution, according to O'Dwyer, as it never happens, the tube always escaping by the mouth.

Nevertheless, a case is on record where the tube was swallowed.

Occasionally, sudden cessation of respiration occurs after the tube has been introduced, caused by false membrane occluding the opening. In that case a small catheter may be passed through the tube to push the membrane out of the way, and while doing this the child will probably die, it is far better to withdraw the tube immediately ; the irritation attendant upon the manipulation usually causes coughing, and false membrane is expelled often in sufficient quantity to relieve the stenosis.

All writers seem to agree upon the position in which the child should be placed while introducing a tube. I do not lay much stress upon this point. I have introduced a tube while the child was lying in bed just as easily as when held by an assistant. Of course, when the operation is done upon a child in bed, you may depend upon it that it is so far gone it cannot resist. The object of the upright position is only for the purpose of holding firmly the struggling child.

What is the result of intubation as compared to tracheotomy ? Waxam has gathered 1027 cases, of which only twenty-six per cent. recovered. Rauchfuss reported to the Deutscher Aerztlicher Verein in St. Petersburg thirty per cent. of recoveries in thirty-eight cases, but adds that in eight cases tracheotomy had to be resorted to. Prescott and Goldwaith report in *Boston Medical Journal* 392 cases of intubation with a mortality rate of seventy-nine per cent. and 139 cases of tracheotomy with a death-rate of 88 per cent. Altogether 2,815 cases of intubation and 23,941 cases of tracheotomy have been collected and analyzed, showing very little difference in the percentage of deaths in the two operations. Although the percentage of recoveries is small, it no doubt would be greater if left alone.

How long may a tube be borne by the larynx, is an interesting question. The longest time I permitted a tube to remain *in situ* was seven days. Dr. I. H. Lynde, of Fillmore avenue, had a patient in which it was left four weeks, but the child finally died. O'Dwyer left his first tubes in thirteen days on an average. In one case it was left in a month, then extracted and re-introduced, remaining in this time over two months without removal. Schmeigelow, of Copenhagen, cites the case of a child six years old, in which intubation was performed three months after tracheotomy; the child finally recovered after having intubation kept up for a whole year. Another case, a child four years old, intubated eight days after tracheotomy, tube removed after a month, cure. A most interesting case is recorded by Dillon Brown, New York. A little girl, aged three and a quarter years, had intubation done seven days after the first appearance of laryngeal symptoms; twelve days afterward, marked dyspnea was present during the course of an attack of diphtheria; dyspnea was immediately relieved, and a piece of pseudo membrane was coughed up. At the end of seven and a half days, the tube was removed, but was returned within fifteen minutes because of urgent dyspnea. Four different attempts were made to remove the tube, but it had to be returned each time after intervals ranging from four hours to thirteen days; it being impossible to insert a full-sized O'Dwyer tube, and the smaller one not relieving dyspnea, tracheotomy was performed. There was marked stenosis of the trachea, not, however, extending along its whole length. At the time of operation, the inner tube only of the canula could be pushed into the trachea, fitting very tightly, but next day the parts being stretched, a regular tube was inserted. A month later, the tracheotomy tube was removed, and after dilating from below with sounds, a three-year-old O'Dwyer tube was inserted. An attack of pneumonia subsequently followed. The tube was removed every month or two, but always had to be inserted within an hour. Digital exploration proved the larynx to be occupied with granulating tissue overlapping the edge of the tube; a larger tube was inserted to press upon the granulations, subsequently this could be removed, and there was no return of the dyspnea. The patient is now perfectly well and has a good, though at times rather harsh, voice. Intubation in this case was continued for nine months.

From the foregoing it appears a tube may be retained any length of time, but it is well to remember that enough irritation may be set up to cause erosion and, finally, granulations which occlude the tube, when removal becomes necessary, probably the larynx dilated, and a larger tube inserted, which will press on the granulations and cause their absorption. In acute stenosis from edema or pseudo membrane, the tube is not left in long enough to produce such an effect. I make it a rule, if the child's condition permits, to remove the tube the third or fourth day and re-insert it if necessary.

The shortest time on record, I think, occurred in one of my cases. I introduced a twelve-year-old tube into the larynx of a ten-year-old girl; it was immediately coughed up, followed by a piece of false membrane two inches long. The child breathed easily afterwards and I declined to replace it that evening. After twenty-four hours, however, I was again called to insert the tube; I removed it the next day, because the girl could take no nourishment. It was not necessary to replace it. This patient was strong and apparently little affected by the disease, but I gave an unfavorable prognosis based upon the frequency of respiration, showing diphtheritic toxemia present. I find whenever respiratory movements reach thirty-five per minute, the case invariably terminates fatally.

All writers agree that it is more difficult to remove a tube than to insert one, and it seems the older the child the more difficult the operation. I found in the girl above quoted, that every effort of inspiration drew the larynx away from my index finger, and it required several attempts before I could locate the opening in the tube. At any rate, it is exceedingly difficult to extract at the first attempt.

In the past year I have intubated seventeen times in twelve children, with four recoveries. The operation was performed in two children on one morning, and each tube removed the third day, both recovered; ages three and four years. Another case, four years old, tube removed fourth day, recovery. The fourth case, a Fitch patient, tube removed the third day; stenosis still very marked, reinsertion refused; a week later I learned the child had recovered. Of the eight cases that died, five had the tube removed several days before death. Dyspnea did not return, death being caused by diphtheritic toxemia. Whether or not all twelve cases were diphtheria, I cannot say, as but two occurred in my own practice, no pseudo membrane being visible and both recovered. The ten other cases occurred in the practice of other physicians; their diagnoses I do not presume to criticize, as all of them had seen a large number of cases, and, I judge, are competent to make a diagnosis, since our best authorities agree to disagree on the question.

My percentage of recoveries is a little higher than the average; those of Dr. Mynter (not including his hospital cases), reach as high as eighty per cent., I am told, in five cases; Dr. Niemand had, of seventeen cases, about forty per cent. of recoveries; Dr.

Park, twenty-five per cent. out of thirty-five cases; Dr. Myers, assistant to Dr. Park, had thirty per cent. out of seventeen cases; Dr. Bergtold obtained thirty per cent. Dr. Renner had thirty-five per cent. So that Buffalo has as good a record as any individual city in the world.

The present O'Dwyer gag, or any other, has given more trouble than is convenient, constantly slipping, and even breaking, under the enormous pressure exerted by the jaws of the child. If a gag could be constructed which would move with the child's head, there would be no danger of slipping and being bitten by the sharp teeth, which occurred to me several times, although just short of penetrating the skin. Such a gag, to be successful, must be self-retaining. In order to run less risk, I had a ring made to fit upon the index finger, between the second and third joints, the most exposed part; it is broad above, and narrow below. The dorsal surface of the finger, being rather thin, is easily penetrated, while the palmar surface being thick, is not affected. Mobility is not lessened by its use, as is the case with other protectors found in the shops.

In conclusion, let me insist on early intubation, not in preference to tracheotomy (for the American people do not take kindly to that operation), but to give the child a very fair chance to recover, if that be possible, for certainly a small proportion die for want of air, and that is always obviated by the tube. The principal objective symptom, among others, which always means obstruction somewhere in the air passage above the bronchi, is a very marked recession at the pit of the stomach at every inspiratory effort, and cannot be overlooked. I make this statement, since surgeons have often been called upon to operate because the patient was breathing thirty or forty times per minute, which, however, had nothing at all to do with the obstruction.

In passing, I may say that intubation is just as efficient in the adult as in the child, and I would never hesitate to use a twelve-year O'Dwyer tube in edema, or any other obstruction of the larynx in the adult, if I had no larger one at hand. I would, however, allow the thread to remain attached to the tube, to prevent slipping. I think it preferable to tracheotomy in such cases, especially when obstruction would not last long anyway; moreover, no scar will be left, as in tracheotomy. Still, in chronic stenosis, it seems to be more serviceable than the tracheotomy tube.

Schmeigelow has performed intubation in six chronic cases He says " Intubation is indicated in all cases of chronic stenosis, and ought, or might, be performed in every case of acute stenosis from diphtheria."

560 MICHIGAN STREET.

